# Method of Relieving Sensitive Dentin

**Published:** 1904-06

**Authors:** I. B. Kenney

**Affiliations:** Park Rapids, Minn.


### METHOD OF RELIEVING SENSITIVE DENTIN.

By I. B. Kenney, D. D. S., Park Rapids, Minn.

In the case of cervical cavities which are always very sensitive, I use a local anesthetic hypodermically exactly as for extracting. Put on dam and dry thoroughly, touch with carbolic acid followed by warm air. Use sharp burs and run engine fast. The adjusting of clamp cannot be felt no matter how far below the gum the cavity extends.

It works splendidly with me and is worth trying on your next case..—*Summary.*

